# Gene therapy restores vision in *rd1* mice after removal of a confounding mutation in *Gpr179*

**DOI:** 10.1038/ncomms7006

**Published:** 2015-01-23

**Authors:** Koji M. Nishiguchi, Livia S. Carvalho, Matteo Rizzi, Kate Powell, Sophia-Martha kleine Holthaus, Selina A. Azam, Yanai Duran, Joana Ribeiro, Ulrich F. O. Luhmann, James W. B. Bainbridge, Alexander J. Smith, Robin R. Ali

**Affiliations:** 1 Department of Genetics, UCL Institute of Ophthalmology, 11-43 Bath Street, London, EC1V 9EL, UK

## Abstract

The *rd1* mouse with a mutation in the *Pde6b* gene was the first strain of mice identified with a retinal degeneration. However, AAV-mediated gene supplementation of *rd1* mice only results in structural preservation of photoreceptors, and restoration of the photoreceptor-mediated a-wave, but not in restoration of the bipolar cell-mediated b-wave. Here we show that a mutation in *Gpr179* prevents the full restoration of vision in *rd1* mice. Backcrossing *rd1* with C57BL6 mice reveals the complete lack of b-wave in a subset of mice, consistent with an autosomal recessive Mendelian inheritance pattern. We identify a mutation in the *Gpr179* gene, which encodes for a G-protein coupled receptor localized to the dendrites of ON-bipolar cells. Gene replacement in *rd1* mice that are devoid of the mutation in *Gpr179* successfully restores the function of both photoreceptors and bipolar cells, which is maintained for up to 13 months. Our discovery may explain the failure of previous gene therapy attempts in *rd1* mice, and we propose that *Grp179* mutation status should be taken into account in future studies involving *rd1* mice.

## Abstract (web summary)

The rd1 mouse is the most widely used model to study retinal degeneration. Here, the authors identify a wide-spread mutation in these mice that may explain the failure of previous gene therapeutic approaches and show that long-lasting restoration of vision is possible in rd1 mice without this mutation.

## Graphical abstract

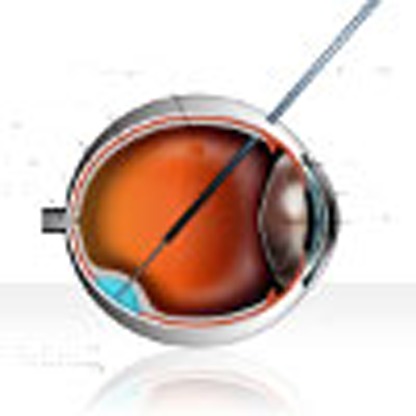

The *rd1* mouse, which was first described in 1924, is considered the first mouse model of retinal degeneration and was initially referred to as the rodless mouse^1^. It carries a naturally occurring nonsense mutation in the photoreceptor cGMP phosphodiesterase 6b (*Pde6b*) gene and is the oldest and most widely used animal model for studying the mechanisms of retinal degeneration. The defective gene encodes the β-subunit of rod PDE (βPDE), an essential component of the phototransduction cascade, a molecular signalling pathway that converts light stimulus into an electric signal in the outer segments of rod photoreceptors^2^,^3^. The rod photoreceptor degenerates rapidly in this model such that by 4 weeks of age, the outer nuclear layer is left with a single layer of photoreceptors consisting of cone photoreceptors^4^ that are subsequently lost through a secondary unknown mechanism. It is thus one of the most severe models of retinal degeneration identified to date.

Because *rd1* mice have been readily available for many decades, numerous attempts have been made to rescue visual function and prevent degeneration in this model. Many attempts have been made through either local or systemic intervention to rescue directly the primary defect in the rod photoreceptors^5^,^6^,^7^,^8^,^9^ and/or slow the secondary degeneration of the cones^10^,^11^,^12^. However, providing effective and lasting phenotypic rescue has proven elusive, the only achievement to date being partial histological preservation with minimal, if any, functional rescue^6^,^7^,^13^,^14^. This has led to the development of a wide-spread notion that the speed of retinal degeneration in this model is too fast to allow a window of opportunity for treatment^14^,^15^,^16^,^17^,^18^,^19^ This hypothesis has been bolstered by an effective gene supplementation therapy in the canine model of PDE6B deficiency (*rcd1* dog)^20^ and in the hypomorphic *Pde6b*-mutant (*rd10*) mouse^15^,^18^,^19^. As photoreceptor loss in these animals is slower, they provide a greater window of opportunity for treatment than the *rd1* mice. These results suggested that the retinal degeneration caused by a defective *Pde6b* gene is in principle amenable to structural and function rescue by gene therapy, but the rate of photoreceptor degeneration in the *rd1* mice is too fast to allow for effective rescue.

Recently, we have provided evidence to challenge the prevailing consensus that the speed of photoreceptor degeneration in *rd1* mice precludes effective rescue by demonstrating effective adeno-associated virus (AAV)-mediated gene supplementation therapy in *Aipl*- deficient mice^21^,^22^. Knockout of the *Aipl1* gene results in post-transcriptional reduction of all the three subunits of the cGMP-PDE holoenzyme (α, ß and γ); in this model the retinal degeneration is even faster than in the *rd1*, resulting in a complete loss of photoreceptors by 3 weeks of age, indicating that the treatment window should be even smaller in the *Aipl1*^−/−^ mouse compared with *rd1 mice*^16^,^23^.

Since the first attempts to rescue the *rd1* mouse, there have been considerable advances in retinal gene therapy, including the availability of new AAV serotypes that allow highly efficient and rapid photoreceptor transduction. Following our successful treatment of *Aipl1−/−* mice we wanted to determine whether an optimized gene therapy could improve the rescue of the *rd1* mice compared with previous attempts. We show that rescue of the functional deficit in the *rd1* mouse by *PDE6B* gene supplementation therapy is compromised by the presence of a confounding mutation in the *Gpr179* gene in most strains. After removal of the *Gpr179* mutation, *PDE6B* gene therapy results in the effective rescue of structure and function of the *Pde6b*^−/−^ retina for up to 1 year.

## Results

### AAV gene therapy restores photoreceptor function in rd1 mice

**Figure 1:**
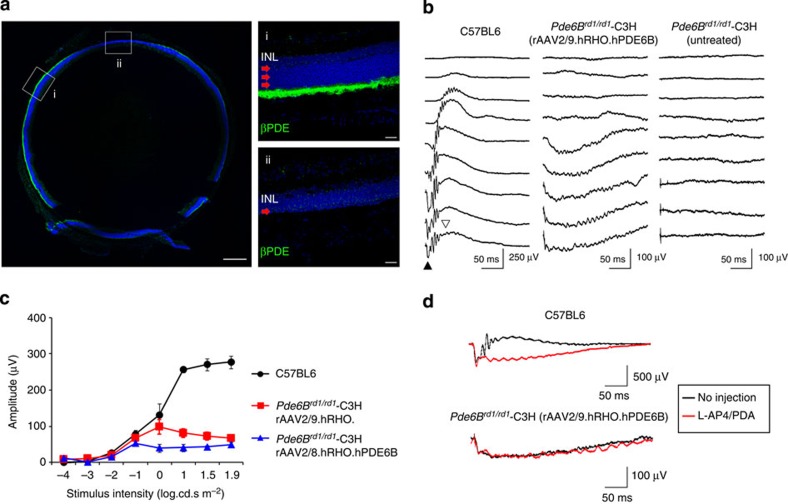
Histological but no functional rescue in Pde6b^rd1/rd1^-C3H mice. (**a**) Presence of βPDE and histological preservation (green) at 2 months post injection following AAV9-mediated gene supplementation in *Pde6b*^*rd1/rd1*^-C3H mice. Red arrows indicate the ONL in treated (i) and untreated areas (ii). Scale bars, 250 (a) and 50 μm (i, ii) (**b**) Absence of b-wave in the eyes of *Pde6b*^*rd1/rd1*^-C3H mice treated with rAAV2/9.hRHO.hPDE6B. Representative ERG traces from C57BL6 mouse and treated and untreated eye from *Pde6b*^*rd1/rd1*^-C3H mice. Filled triangle indicates a-wave and open triangle denotes b-wave. (**c**) Comparison of a-wave amplitudes between the eyes with AAV8- or AAV9-mediated expression of βPDE in *Pde6b*^*rd1/rd1*^-C3H mice (*n*=4 per group) and C57BL6 (*n*=5). Data represent mean±s.e.m. (**d**) Absence of bipolar response indicated by pharmacological blockage of the synaptic connection between photoreceptors and bipolar cells in *Pde6b*^*rd1/rd1*^-C3H mouse. Black traces represent recording before injection of L-AP4 and PDA. Red traces indicate responses recorded from the same animal after intravitreal injection of L-AP4 and PDA that blocks the synaptic transmission between photoreceptor and bipolar cells. Note that waveform remains relatively similar after the injection in the gene therapy-treated C3H mouse (lower traces), whereas a large reduction of the positive peak (b-wave) is seen following the injection in C57BL6 mouse (upper traces).

An AAV2/9 vector carrying the human *PDE6B* gene under the control of human rhodopsin promoter (rAAV2/9.hRHO.hPDE6B) was injected subretinally at postnatal day 9 (P9) in female *rd1* mice on the C3H/HeN background (*Pde6b*^*rd1/rd1*^-C3H). At 2 months of age (~7 weeks post injection), histological evaluation of the treated eyes showed substantial preservation of the outer nuclear layer (ONL) and inner/outer segment structures that corresponded well with the area immunopositive for βPDE (Fig. 1a). Conversely, outside the area of βPDE immunepositivity the ONL was severely reduced to a single layer, consistent with the known progress of retinal degeneration in *rd1* mice^4^. These results confirmed that AAV9-mediated *PDE6B* gene transfer results in the expression of βPDE in rod outer segments and subsequent prevention of rod photoreceptor death. We observed a similar result following the administration of an AAV2/8 vector carrying the same construct (Supplementary Fig. 1).

Electroretinography (ERG) performed at 7 weeks post injection in the rAAV2/9.hRHO.hPDE6B-treated group, revealed that the morphological preservation of the retina was accompanied by a partial functional rescue of the a-wave, which represents rod photoreceptor function. However, the b-wave, initiated by the bipolar cells, was completely absent (Fig. 1b,c). Similar results were also obtained when *PDE6B* gene was delivered using AAV2/8 vector (Fig. 1c). The presence of an a-wave indicated the successful restoration of βPDE activity and a functional phototransduction cascade. Nonetheless, the lack of b-wave indicated either a major defect in the synaptic transmission between the photoreceptors and the immediate downstream bipolar cells or an intrinsic dysfunction of the bipolar cells themselves. This b-wave dysfunction is reminiscent of the pharmacological blockade of photoreceptor-to-bipolar transmission, where the injection of a L-(+)-2-amino-4-phosphonobutyric acid (L-AP4) and cis-2,3-piperidine dicarboxylic acid (PDA) cocktail into the eyes of wild-type (WT) C57/B6 mice abolishes the b-, but not the a-wave^24^,^25^. Indeed, ERG traces before and after pharmacological blockade showed essentially no change in the waveform in the rAAV2/9.hRHO.hPDE6B-treated eyes of *Pde6b*^*rd1/rd1*^-C3H mice (Fig. 1d). These results indicate that, despite the successful histological preservation of the retina following gene supplementation of *PDE6B*, the rod bipolar cells were non-responsive following the restoration of photoreceptor function.

### Bipolar dysfunction in rd1 mice is caused by a Gpr179 mutation

**Figure 2:**
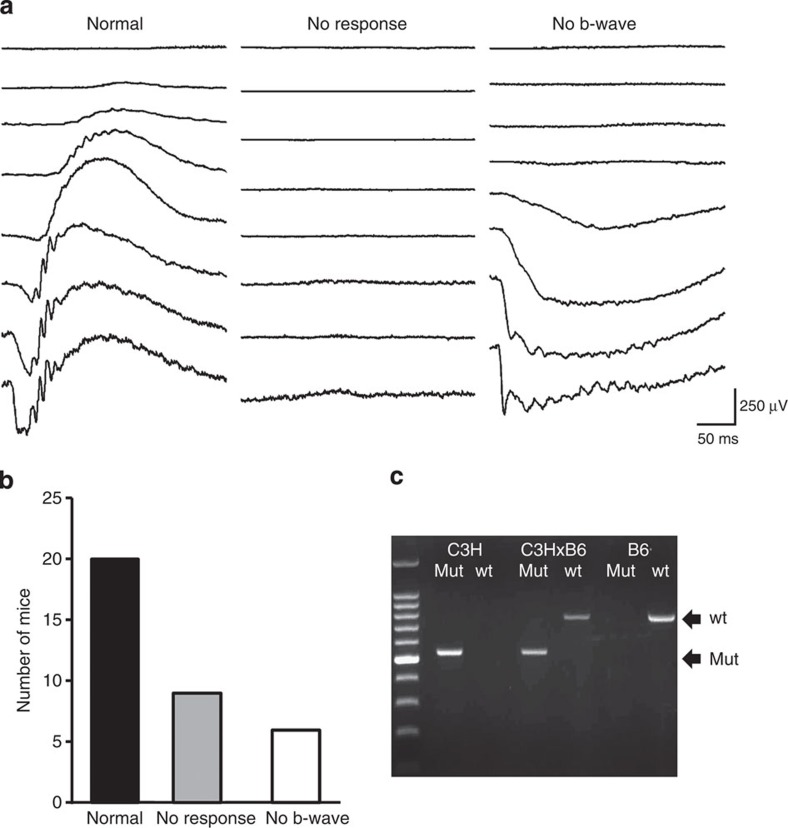
A Gpr179 mutation leads to bipolar dysfunction in the Pde6b^rd1/rd1^-C3H line. (**a**) Three different ERG phenotypes observed in F2 backcross of *Pde6b*^*rd1/rd1*^-C3H and C57BL6. Note that in addition to mice with normal ERGs (left traces), those with no detectable responses (middle traces) and those lacking b-wave in the presence of a-wave were observed. The ‘no b-wave’ ERGs were similar to the ERG waveform observed in *Pde6b*^*rd1/rd1*^-C3H mice treated with rAAV2/9.hRHO.hPDE6B. (**b**) Results of ERG phenotyping of 35 mice of F2 backcross. Wild-type ERG was by far the most frequent phenotype, while the ‘no response’ and ‘no b-wave’ phenotype were similar in number. (**c**) Identification of the homozygous mutations in the *Gpr179* gene in the C3H line. Amplified PCR fragments show the presence of the intronic insertion in the *Gpr179* gene. The results were consistent with *Pde6b*^*rd1/rd1*^-C3H mice carrying homozygous *Gpr179* mutations.

βPDE is a specific component of the phototransduction cascade, which takes place exclusively at rod photoreceptor outer segments. Therefore, the absence of a bipolar cell response in the treated *Pde6b*^*rd1/rd1*^-C3H eyes with restored rod function was an unexpected result. We reasoned that one of the most straightforward explanations would be the existence of an as yet unknown genetic defect in the bipolar cells of the *rd1*-C3H/HeN line. To test this hypothesis, we crossed *Pde6b*^*rd1/rd1*^-C3H with C57BL6 and then crossed the F1 animals. ERG recordings of female F2 offspring, performed at 3 months of age, showed three distinct ERG phenotypes: a normal ERG, as seen in wild-type mice; a non-detectable ERG typical for *rd1* mice and an ERG with no b-wave that resembled the traces of the treated *Pde6b*^*rd1/rd1*^-C3H mice (Fig. 2a). The results of the phenotypic screening of 35 F2 animals were consistent with an autosomal recessive Mendelian inheritance pattern for an absent b-wave; 17% of animals had an absent b-wave; 25% had an absent ERG (presumably *Pde6b*^*rd1/rd1*^genotype) and 57% had a normal ERG (Fig. 2b).

At least four naturally occurring mice lines, that are, *nob1*, *nob2*, *nob3* and *nob5*, have been reported to harbour a similar ‘no b-wave’ or ‘nob’ phenotype^26^,^27^,^28^,^29^. Through a literature search, we noted that one of these lines (*nob5*), in which rod and cone ON-bipolar cell function is absent, was originally derived from the C3H line. As it was plausible that the *Pde6b*^*rd1/rd1*^-C3H line could carry the same mutation, a large intronic insertion in the *Gpr179* gene, we investigated its presence in our mice. Indeed, PCR analysis of *Pde6b*^*rd1/rd1*^-C3H genome confirmed the homozygous intronic insertion in the *Gpr179* gene to be present in the mice with a ‘no b-wave’ phenotype (Fig. 2c). Furthermore, the genotype of 27 F2 and 62 F3 animals was confirmed by PCR and showed the expected correlation with ERG recordings: animals without an ERG were homozygous for the *Pde6b* mutation, animals without a b-wave were homozygous for the *Gpr179* mutation and animals with normal ERGs had at least one wild-type allele for both the genes. The *Gpr179* mutation was also identified in *Pde6b*^*rd1/rd1*^-C3H mice from an alternative independent supplier (Supplementary Fig. 2).

### Functional rescue in rd1 mice without the Gpr179 mutation

**Figure 3:**
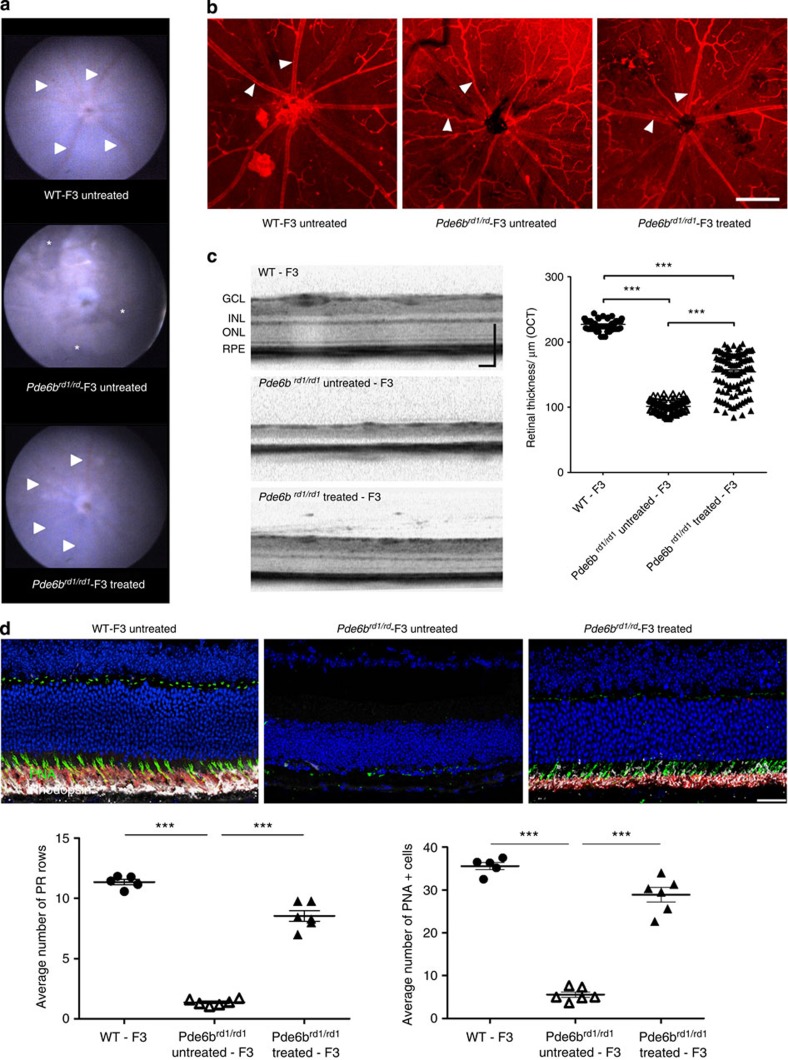
Preservation of structure after AAV-mediated gene therapy in Pde6b^rd1/rd1^-F3 mice. (**a**) Fundus photos from a treated and an untreated eye of *Pde6b*^*rd1/rd1*^-F3 mouse and a normal eye of a WT-F3 control. Note that retinal vessels (arrowheads) are clearly visible in the treated (bottom) *Pde6b*^*rd1/rd1*^-F3 mouse and WT-F3 mouse (top), whereas vessels in the untreated eye are not very visible (middle). Instead, areas of pigmentation (asterisks) are visible in the untreated eye of *Pde6b*^*rd1/rd1*^-F3 mouse. (**b**) Retinal flatmount stained with isolectin B4. Note retinal vessels (arrowhead) are severely attenuated in the untreated *Pde6b*^*rd1/rd1*^-F3 retina (middle panel) compared with the treated (right panel) or the WT-F3 control retina (left panel).Scale bar,250 μm. (**c**) OCT images from a treated and an untreated eye of *Pde6b*^*rd1/rd1*^-F3 and normal eye of a WT-F3 control. Quantification of retinal thickness: individual measurements obtained from six animals (*n*=5 for WT control) are shown. ***P<0.0001 One-way analysis of variance (ANOVA) with Tukey’s multiple comparison test. Data represent mean±s.e.m. (**d**) Histological retinal sections from the mid-central retina of a treated and an untreated eye of *Pde6b*^*rd1/rd1*^-F3 and a normal eye of a WT-F3 control. Quantification of number of DAPI labelled photoreceptor (PR) rows (left) and number of cones per section (PNA+ cells—right). ***P<0.0001 One-way ANOVA with Tukey’s multiple comparison test, *n*=5 for control, *n*=6 for treated and untreated animals. Data represent mean±s.e.m. Scale bar, 50 μm.

After establishing an F3 backcross of *rd1* mice homozygous for the *Pde6b* mutation, but devoid of the *Gpr179* mutation (*Pde6b*^*rd1/rd1*^-F3), rAAV2/9.hRHO.hPDE6B was injected subretinally in female P9 pups. At 13 weeks after the treatment, fundus photography showed reduced pigment deposition, suggesting a reduction in photoreceptor death, and larger retinal vessels, possibly reflecting greater demands of nutrients in the retina compared with the untreated contralateral eye (Fig. 3a). The difference in vascular diameter was also readily recognizable on retinal flatmounts stained with the blood vessel marker isolectin B4. The retinal vasculature in the treated eye was very well preserved compared with the severe vascular attenuation in the untreated eye (Fig. 3b). Optical coherence tomography (OCT) imaging showed the presence of the ONL in the treated eyes, in contrast with the untreated eyes that lacked a visible ONL (Fig. 3c). The average thickness of the whole retina in treated eyes was 154±30 μm, which was equivalent to ~70% (227±7 μm) of that observed in wild-type control mice devoid of both *rd1* and *Gpr179* mutations (WT-F3 mice) established during the process of the backcross (Fig. 3c). Histological sections from the mid-central retina confirmed the *in vivo* observation, showing significant preservation of the photoreceptors (Fig. 3d). Quantitative analyses showed increased number of rows of photoreceptor nuclei in treated eyes (mean±s.d.=9±1 rows of photoreceptors) compared with untreated eyes (1±0), corresponding to 80% of wild-type controls (11±1). The number of cone photoreceptors identified by peanut agglutinin (PNA) staining was also increased in the treated eyes compared with untreated, corresponding to 80% of wild-type controls (Fig. 3d).

**Figure 4:**
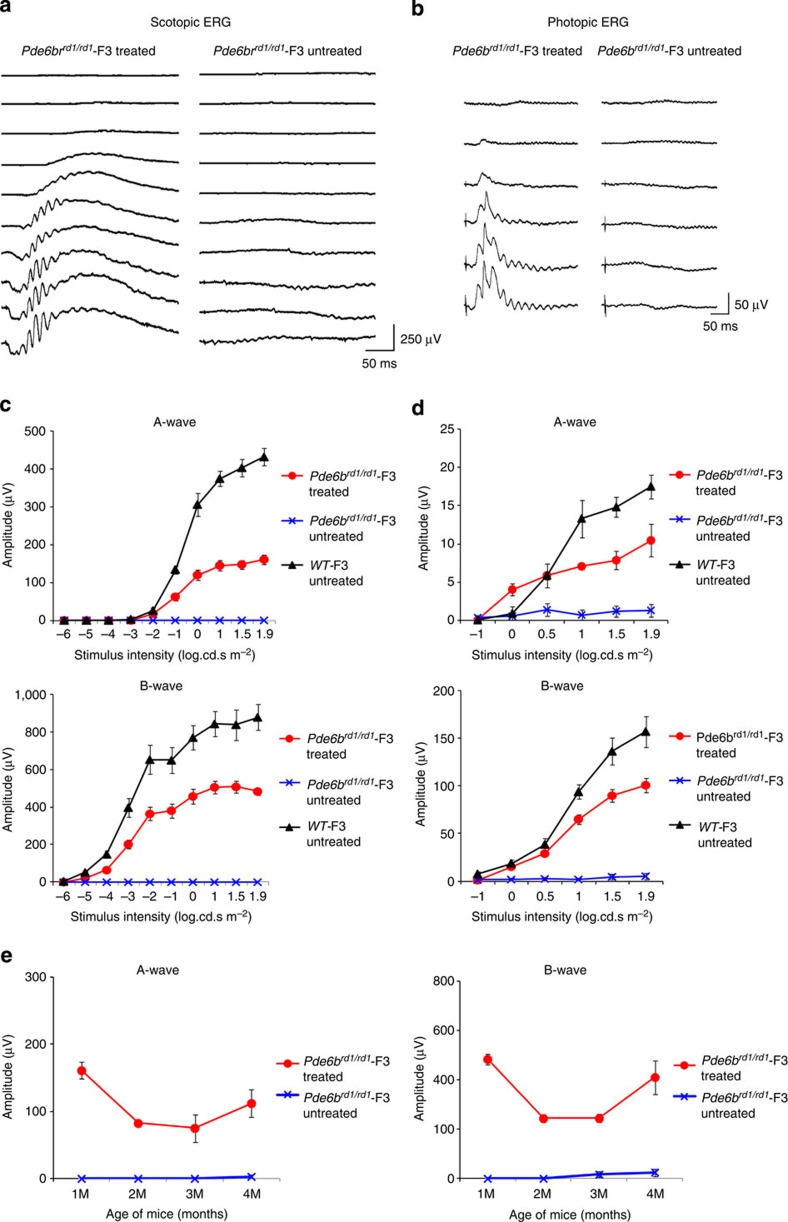
Restoration of photoreceptor and bipolar cell function in Pde6b^rd1/rd1^-F3 mice. (**a**–**d**) Scotopic and photoptic ERGs at 3 weeks following gene transfer. Representative ERG traces from treated and untreated eyes from a single *Pde6b*^*rd1/rd1*^-F3 mouse for scotopic (**a**) and photopic (**b**) flashes. Quantification of the ERG amplitudes in treated (*n*=6) and untreated eyes (*n*=6) from *Pde6b*^*rd1/rd1*^-F3 and WT-F3 (*n*=5) mice for scotopic (**c**) and photopic (**d**) flashes. (**e**) Relatively stable scotopic a-wave and b-wave ERG responses 4 months after treatment (1.9 log cd s^−1^ m^−2^; *n*=6). Data points and error bars in the plots **c**,**d** and **e** represent mean±s.e.m.

The severe and rapid photoreceptor degeneration in *rd1* mice results in a complete absence of ERG responses, including cone-mediated ones, at 1 month after birth^4^. At 3 weeks after the treatment (~1 month after birth), we observed a substantial retinal response in *Pde6b*^*rd1/rd1*^-F3 mice. Both a- and b-waves were clearly visible, confirming the restoration of both photoreceptor and bipolar cell function. The treated eyes, at the brightest flash intensity under scotopic conditions, had a-wave and b-wave amplitudes that were 37% and 55%, respectively, of the ERG amplitudes from isogenic control mice (WT-F3; Fig. 4a,c). Meanwhile, the untreated eyes from *Pde6b*^*rd1/rd1*^-F3 mice showed no detectable responses. Gene therapy restored normal rod sensitivity, as determined by the dimmest light intensity at which the retinal response emerged under scotopic conditions (Fig. 4a,c). ERG recordings under photopic conditions also demonstrated the preservation of cone photoreceptor function. Treated animals, at the brightest flash intensity, had cone ERG a-wave and b-waves amplitudes that were 60% and 64%, respectively, of that of WT-F3 mice (Fig. 4b,d). Although there was some reduction in ERG amplitudes, particularly between the first 3 weeks and the second month after injection, they remained stable thereafter until at least 4 months after treatment (Fig. 4e,f).

### Restoration of visually-guided behaviours and visual acuity

**Figure 5:**
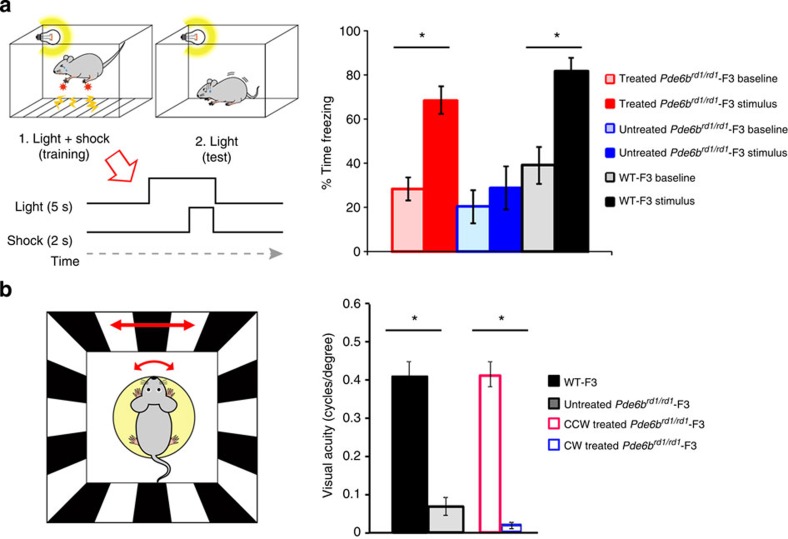
Supplementation of PDE6B gene to rods restores retinotopy and visual function. (**a**) Visually cued fear conditioning paradigm. On the day of training, each mouse was placed in a conditioning chamber and received six presentations of a 5-s flickering light stimulus paired with a co-terminating 2-s foot shock (left panel). Recall of light-cued memory was tested 24 h post training by measuring the baseline and light-cued freezing levels in a novel environment (left panel, see methods). Rescue of visually cued behaviour in PDE6B treated mice (right panel). Bar graph represents mean average of the percentage of time spent freezing during a baseline period without light stimulation (outlined bars) and during presentation of the light cue (filled bars). Light-cued memory was assessed for three different experiment groups; *Pde6b*^*rd1/rd1*^-F3 treated (red bars, *n*=6), *Pde6b*^*rd1/rd1*^-F3 untreated (blue bars, *n*=5) and untreated wild-type-F3 (black bars, *n*=4). The experiment was conducted at 10 weeks after treatment. (**b**) Restoration of normal visual acuity in the treated eyes of *Pde6b*^*rd1/rd1*^-F3 mice. Note that when visual acuity in response to CCW direction representing the function of the treated eyes and to clockwise direction probing that of the untreated eyes separately within the same *Pde6b*^*rd1/rd1*^-F3 animal (*n*=6), treated eyes showed comparable acuity to those of the WT-F3 controls (*n*=5), whereas untreated eyes showed similar values to the untreated *Pde6b*^*rd1/rd1*^-F3 mice (*n*=5). The experiment was conducted at 6 weeks after treatment. Data represent mean±s.e.m. Statistical significance was assessed with a one-way analysis of variance, * denotes *P* value<0.05. Data represent mean±s.e.m.

To test whether restoration of retinal function by gene therapy translates into improved vision, we used two different methods to assess visually-guided behaviour. First, we analysed the behaviour of female mice (freezing their motion) following fear-conditioned learning, where the visual cues were paired with a mild foot shock 10 weeks after the injection of the vector. Treated *Pde6b*^*rd1/rd1*^-F3 mice showed significantly increased levels of freezing behaviour upon presentation of the visual cue (baseline freezing, 28.5±5.2; light-cued freezing, 68.4±6.2; mean±s.e.m.; *P*<0.01, Fig. 5a, red bars) indicating that they had learned the association between the light cue and the foot shock. The increase in freezing behaviour was comparable to that measured in WT-F3 mice (baseline freezing, 39.1±8.3; light-cued freezing, 81.6±6.3; mean±s.e.m; *P*<0.05 Fig. 5a, black bars). In contrast, untreated *Pde6b*^*rd1/rd1*^-F3 did not demonstrate a significant increase in freezing behaviour during cue presentation (baseline freezing, 20.3±7.6; light-cued freezing, 28.8±10.0; mean±s.e.m; Fig. 5a, blue bars).

To further investigate the quality of vision that was restored, visual acuity was measured by tracking head movements in response to horizontally moving sinusoidal grating using Optomotry 6 weeks after injection of vector under the brightest test condition possible (62 cd m^−2^). The untreated *Pde6b*^*rd1/rd1*^-F3 mice had significantly worse visual acuity (0.08±0.02 cycles per degree; mean±s.e.m.) compared with the age-matched WT-F3 mice (0.41±0.04 cycles per degree; mean±s.e.m.). Following treatment, an improvement was observed for the treated right eye (counter-clockwise (CCW) direction; 0.41±0.03 cycles per degree; mean±s.e.m.) but not for the untreated eye (clockwise direction; 0.02±0.00 cycles per degree; mean±s.e.m.), indicating near-normal visual acuity in the treated eye, whereas the acuity of the untreated eye remained at background levels (Fig. 5b).

### Long-term preservation of vision after AAV gene therapy

Examination of eyes, 13 months after treatment with rAAV2/9.hRHO.hPDE6B, showed the presence of a substantial photoreceptor layer with visible inner and outer segment structures while no discernable photoreceptors were visible in the untreated eyes (Supplementary Fig. 3). Immunohistochemical assessment confirmed that the treated photoreceptors expressed rhodopsin and the βPDE in the outer segments. This was accompanied by substantial ERG responses at 11 months with clearly visible scotopic a-wave and b-wave components (Supplementary Fig. 4A,B). Although visual acuity was reduced at 11 months compared with the early time points, it was still substantial and significantly higher than in untreated eyes (Supplementary Fig. 4C).

## Discussion

This study demonstrates, for the first time, the unambiguous structural and functional rescue of rod photoreceptors in *rd1* mice, the most widely studied model of retinal degeneration. We have shown that simple gene transfer of the defective gene, *Pde6b*, into the commonly used laboratory *rd1* strain (C3H/HeN) can rescue rod photoreceptor function, but fails to restore the downstream signalling. We discovered that this is explained by the presence of an additional naturally occurring mutation in the *Gpr179* gene within the same line that abolished the function of retinal ON-bipolar cells, including rod bipolar cells. Indeed, the restoration of both photoreceptor and bipolar function was only possible after crossing this mutation out from the C3H/HeN mouse line.

C3H mice lines with the *rd1* mutation are widely distributed for research purpose by most major animal suppliers worldwide. Indeed, a review of over 130 research articles, where the *rd1* background was stated, shows that 67% of these studies use the C3H line. Our investigations suggest that the *Gpr179* mutation may be present in many C3H lines, including those provided by the Jackson Laboratory, Charles River and Harlan Laboratories. The spontaneous mutation in the *Gpr179* gene was first identified as the cause of nob5 mice, a strain that originated from the C3H line bred by the Jackson Laboratory^29^. We have directly confirmed by PCR the presence of the homozygous *Gpr179* mutation in the C3H/HeN line distributed by both Charles River and Harlan Laboratories. Historically, the Jackson Laboratory received C3H mice from Heston in 1948 (C3H/HeJ strain) while Charles River received C3H mice from the National Institute of Health (NIH, USA) in 1974, which were originally provided to the NIH by Heston in 1951 (C3H/HeN strain)^30^. Thus, the *Gpr179* mutation was likely already present at the time the line was propagated to the Jackson Laboratory by Heston in 1948. This would indicate that most, if not all, of the studies using *rd1*-C3H mice as a model of retinal degeneration published within the past 65 years (since 1948) probably used the double mutants unknowingly and that the conclusions, based on the erroneous assumption that these mice are a simple model of photoreceptor degeneration, should be interpreted with caution. In particular, since *Gpr179* expression is specific to ON-bipolar cells^29^, any rescue of vision in this mouse line must be limited to the OFF cone bipolar pathway. This also raises the question of how approaches such as transplantation of rod precursors in C3H/HeN mice can lead to the efficient rescue of vision as recently reported by Singh *et al*.^31^. Considering the lack of function in the canonical ON rod bipolar pathway, the extensive contacts observed between the transplanted rods and rod bipolar cells cannot explain the reported rescue of vision. This would require a more complex explanation, such as the extensive wiring between rods and OFF cone bipolars, although to date this has not been reported.

We cannot fully exclude that a mutation in another gene, closely linked to the *Gpr179* gene, may be responsible for the observed phenotype. However, in view of the similarity with the disease phenotype of GPR179-deficient patients, mice and zebrafish^29^, we consider this unlikely. The exact impact of the large intronic insertion in *Gpr179* is unknown. Since a targeted sequencing of the entire *Gpr179* gene identified no other mutations^29^, it is likely that the large intronic mutation affects the production of the encoded protein. As it does not directly affect the coding exons and because its mRNA was virtually absent in the retina^29^, it is likely that the mutation results in the aberrant splicing of the gene followed by a nonsense-mediated decay of the mRNA. Irrespective of the actual mechanism, our findings show that when using *rd1* mice on the C3H background as a model of photoreceptor degeneration it is essential to ensure that the mice are free of the Gpr179 mutation. The results presented here, together with the recent identification of the rd8 mutation in many transgenic mouse lines^32^,^33^, highlight the risks of using inbred mouse strains that potentially carry mutations in more than one gene. Although our work does not necessarily invalidate the results of previous studies using rd1 mice, the increasing emphasis on functional outcomes and vision means that future studies using this animal model might be severely compromised if the Gpr179 status of the animals is not taken into account.

In addition to the robust structural rescue and restoration of the retinal circuit, treated mice also showed vision-guided behaviour that was absent in untreated animals. The most intriguing outcome was the demonstration of normal visual acuity in the treated eye that contrasted sharply with the completely blind contralateral untreated eye. This is a level of therapeutic benefit that has not been demonstrated by previously reported gene supplementation therapies^6^,^7^,^8^,^13^,^14^. Of these previous attempts, the *PDE6B* cDNA iontophoresis study by Souied *et al*.^8^ achieved the best results. They appear to use the *rd1* mice on a C57BL/6 background and achieved a morphological rescue of about 45% of wild-type ONL thickness; it was also the only study to report a small ERG recovery of about 10% of wild-type levels. In this study, however, we were able to achieve an 80% rescue of ONL thickness and an ERG recovery of around 60% compared with the wild-type controls. The improved rescue reported here could be explained by several factors including the use of more efficient gene delivery tools, the use of a strong rod-specific promoter and the age at which the animals were treated. Both AAV8 and AAV9 have been shown to be very efficient in targeting photoreceptors and are capable of delivering high levels of transgene expression. In this study, we established that the two vectors are capable of delivering similar levels of functional and morphological rescue (data not shown). While the treated eyes in this study showed some decline in retinal function during the year following injection, none of the previous studies were able to demonstrate substantial morphological rescue beyond 6 weeks post treatment. This study demonstrates, for the first time, a robust, long-term structural, functional and behavioural rescue of blind *rd1* mice and supports the development of gene therapy for retinitis pigmentosa caused by mutations in *PDE6B.*

## Methods

### Animals

The *rd1* mice (C3H/HeN) and wild-type mice (C57BL/6J) were purchased from Harlan Laboratories (Blackthorn, UK). Additional *rd1* mice were purchased from Charles River (Margate, UK). All mice were maintained under cyclic light (12 h light–dark) conditions; cage illumination was 7 foot-candles during the light cycle. All experiments were approved by the local Institutional Animal Care and Use Committees (UCL, London, UK) and conformed to the guidelines on the care and use of animals adopted by the Society for Neuroscience and the Association for Research in Vision and Ophthalmology (Rockville, MD, USA). Animal group sizes were based on power calculations (power=0.9, *α*=0.05).

### Backcrossing and genotyping

Several homozygous *rd1* female mice were paired with C57BL/6J wild-type male mice. The heterozygous litters from this crossing (F1) were then paired with each other. The resulting litters (F2) from the F1 heterozygous pairing were phenotyped by ERG, PCR and DNA sequencing. Ear clips were collected from all F2 litters and the genomic DNA (gDNA) was extracted using the DNAreleasy (Anachem, Luton, UK) following the manufacturer’s protocol. Extracted gDNA was used for PCR amplification of *Gpr179* and *Pde6b* gene fragments using GoTaq Green (Promega, UK) as follows: 12.5 μl of GoTaq Green Master Mix, 1 μl of each primer at 10 μM, 2 μl of gDNA and water up to a total of 25 μl. Primers used are shown in Supplementary Table 1 and cycling conditions in Supplementary Table 2. For the *Gpr179* genotyping separate reactions were set up for the wild-type and for the mutant allele screening using different reverse primers but the same forward primer (GPR179 F1). Since the *rd1* allele has a point mutation, the genotype was determined by sequencing the PCR product. The sequence for the GPR179 R3, which binds to the mutant allele only, was defined after using the GPR179 F1 primer to sequence intron 1 and define the exact position and partial sequence of the large insertion that is responsible for the non-functional *Gpr179* gene in these animals.

### Plasmid constructs, viral production and injection procedure

The transgene construct (pD10/hRho-hPDE6B) was kindly provided by Professor Alberto Auricchio (TIGEM Institute, Naples, Italy) and contains the human cDNA sequence of the *PDE6B* gene downstream of the short human rhodopsin promoter described previously^33^. The plasmids were packaged into AAV8 and AAV9 to generate two recombinant AAV viral vectors, AAV8.hRho.hPDE6B and AAV9.hRho.hPDE6B, as described below (ref. 34). Recombinant AAV8 vector and AAV9 vector were produced through a triple transient transfection method. The plasmid construct (pD10), AAV serotype-specific packaging plasmid and helper plasmid, in a ratio of 1:1:3 at 20 μg total DNA per ml of DMEM, were mixed with Polyethylenimine (Polysciences Inc.) to a final concentration of 50 μg ml^−1^ and incubated for 10′ at room temperature to form transfection complexes that were added to 293 T cells at 50 μg DNA per 15-cm plate and left for 72 h. The cells were collected, concentrated and lysed by freeze–thaw (3x) in PBS to release the vector. AAV8 was bound to an AVB Sepharose column (GE Healthcare), and eluted with 50 mM Glycine pH2.7 into 1 M Tris pH 8.8. AAV9 was purified by size separation on a Sephacryl S300 column, followed by anion exchange chromatography using a POROS 50 HQ column, eluting the vector in 20 mM bis-tris propane, 20 mM Trizma Base and 0.24 M NaCl pH9. Vectors were washed in 1 × PBS and concentrated to a volume of 100–150 μl using Vivaspin 4 (10 kDa) concentrators. Viral particle titres were determined by comparative dot-blot DNA prepared from purified viral stocks and defined plasmid controls. Purified vector titres used for all experiments were 2 × 10^12^ viral particles per ml^−1^. Subretinal injections were performed under direct retinoscopy thorough an operating microscope. The tip of a 1.5-cm, 34-gauge hypodermic needle (Hamilton) was inserted tangentially through the sclera of the mouse eye, causing a self-sealing wound tunnel. The needle tip was brought into focus between the retina and retinal pigment. Animals received double injections of 1.5 μl each to produce bullous retinal detachments in the superior and inferior hemisphere around the injection sites. Eyes were assigned as treated and (contralateral) control eyes using a randomization software.

### Electroretinogram (ERG)

ERGs were recorded from both eyes from mice with and without *Pde6b*^rd1/rd1^ mutation. All animals were dark adapted overnight before ERG recordings. After dilating the pupils using 2.5% phenylephrine and 1.0% tropicamide, ERGs were recorded using commercially available equipment (Espion E2, Diagnosys, LLC, MA). Scotopic recordings were obtained from dark-adapted animals at the following increasing light intensities: 0.0001, 0.001, 0.01, 0.1, 1, 10, 31.2 and 75.2 cd s^−1^ m^−2^ for 8-step protocol and additional 2 flash intensities (0.000001 and 0.00001) for 10-step protocol. Photopic recordings were performed following 5 min light adaptation intervals on a background light intensity of 30 cd m^−2^, which was also used as the background light for the duration of photopic recordings. Photopic light intensities used were 0.01, 0.1, 1, 10, 31.2 and 72.5 20 cd s^−1^ m^−2^. In a small subset of wild-type and C3H/HeN mice, ERGs were recorded after 2 μl of PBS with 20 mM L-AP4 (l-2-amino-4-phosphobutyric acid; Abcam) and 100 mM 2,3 cis-PDA(Abcam) were injected intravitreally into the eye, which has been previously shown to stably abolish the functional connection between photoreceptors and bipolar cells for at least 24 h (ref. 35).

### Fundus photography and optical coherence tomography (OCT)

Fundus photos were obtained using topical endoscopic fundus imaging as previously described^36^. A 5-cm endoscope 3 mm in outer diameter (1218 AA; Karl Storz, Tuttlingen, Germany), was connected by fibre-optic cable to a Nikon D300s digital camera with a 12.3 megapixel charge-coupled device sensor and Viscotears (Novartis Pharmaceuticals, UK) was used as a coupling agent for corneal contact. OCT images were obtained using the Spectralis HRA+OCT (Heidelberg engineering, Heidelberg, Germany) using the 30° angle lens to determine the thickness of the retina *in vivo*. For quantification, four b-scans (50 individual scans averaged) were obtained at standardized position (about 2–3 disc diameters away from the optic disc) in the superior, inferior (both horizontal scans), nasal and temporal (both vertical scans) retina. The OCT images were exported and processed in Adobe Photoshop CS 2 (Adobe Systems Incorporated, San Jose, USA). The processed b-scans were imported into the Fiji image processing software^37^ and scaled by the vertical scale bar. Five thickness measures at equidistant positions across each b-scan were obtained resulting in a total of 20 individual measures per eye. Statistical analyses were performed using GraphPad Prism 5 for Windows (GraphPad Software Inc, La Jolla, USA).

### Immunohistochemistry

Animals were killed, the eyeballs enucleated and cornea, lens and iris removed. For retinal sections, the eyecups were fixed in 4% paraformaldehyde for 1 h and incubated in 20% sucrose for 1 h, all at room temperature, before embedding in optimal cutting temperature medium. 18-μm cryosections were cut in sagittal orientation, rinsed with PBS and blocked in 10% normal goat serum, 3% bovine serum albumin and 0.1% Triton-X100. Retinal flatmounts were dissected during the 1-h fixation period in 4% paraformaldehyde and blocked with 5% normal goat serum, 1% bovine serum albumin and 3% Triton-X100. The respective samples were incubated with primary antibodies in block solution at 4 °C overnight using rabbit anti-PDE6β (diluted 1:500; PA1-722,Thermo Scientific), mouse anti-rhodopsin (diluted 1:1,000; Clone RET-P1, O4886, Sigma Aldrich), lectin PNA Alexa488 conjugate (diluted 1:200; L21409, Life Technologies) and biotin conjugated Isolectin B4 (BSI-B4, L2140, Sigma). Following PBS washes, the respective combination of secondary antibodies (all diluted 1:500, Life Technologies) including goat anti-rabbit Alexa Fluor 546 (A11035), goat anti-mouse Alexa Fluor 633 (A21052) and streptavidin, Alexa Fluor 633 conjugate (S21375) were used to label the samples before these were counterstained with 4',6-diamidino-2-phenylindole (DAPI) and mounted with DAKO fluorescent mounting media (DAKO, S3023, Denmark). Images were acquired by confocal microscopy (Leica DM5500Q). For ONL thickness quantification the number of rows of DAPI-positive nuclei in the ONL were counted on single-layer confocal images taken from the superior and inferior mid-central retina of each eye and averaged per animal. For quantification of cone outer segment the presence of PNA-positive cells were counted on corresponding standardized sized projection images. The average per animal is derived from all the values obtained from three sections per eye with two images each, one from the inferior and one from the superior retina. One-way analysis of variance (Mean±s.d.) followed by Bonferri post-test was performed to determine the significance.

### Fear conditioning

Mice were trained and tested using a commercially available fear conditioning system (Med Associates). To ensure blind conditions, the experimenter performing the training and testing was always blind to the strain of mouse and treatment conditions. In brief, the setup consisted of a conditioning chamber (20 × 30 cm) with a stainless steel grid floor placed inside a sound-attenuating cubicle. Mouse behaviour was monitored constantly during training and testing by means of a built-in infrared digital video camera (30 frames per s^−1^ acquisition rate) and infrared illumination. VideoFreeze software (Med Associates) was used to control the delivery of the LED light stimulus (4.49 log cd s^−1^ m^−2^) with a diffuser in front and shock. The light stimulus consisted of a single LED (535 nm, Thorlabs) 5-Hz 50-ms flicker generated via an arduino interface (Arduino Software) positioned on a side panel of the conditioning chamber. To ensure that the context in which training and testing took place were different, floor and curved wall panels were inserted into the chamber for the testing session.

Mice were placed inside the chamber and underwent one conditioning session, consisting of six pairings of a 5-s light stimulus that co-terminated with a 2-s 0.65 mA foot shock. Inter-trial interval was pseudo-randomized (average interval 90 s). Following the training session mice were returned to the home cage. Twenty-four hours after training, mice were tested for visually cued memory recall. Mice were placed in the test chamber and monitored for a total of 360 s. The conditioning light stimulus was presented continuously for the last 120 s of the test session. All data was acquired and scored automatically by VideoFreeze software (Med Associates). In brief, the software is calibrated before placing the animal in the chamber. The software then measures the pixel changes that take place between every video frame. The motion threshold was set to be as low as possible (20 motion index units), and the continuous freezing count was set to the frame rate to ensure the most sensitive read-out of motion. To assess light-cued memory recall the percentage time of freezing behaviour was averaged for the 2 min immediately before and following the light stimulus onset. Statistical significance was assessed with a one-way analysis of variance. Results are presented as mean±s.e.m.

### Optomotry

Visual acuities were measured by observing the optomotor responses of mice to rotating sinusoidal gratings (OptoMotry, Cerebral Mechanics)^38^. The protocol used yields independent measures of the acuities of right and left eyes based on the unequal sensitivities of the two eyes to pattern rotation as only motion in the temporal-to-nasal direction evokes the tracking response. As a result, the right and the left eyes are most sensitive to CCW and clockwise rotations, respectively^39^. A double-blind two-alternative forced choice procedure was employed, in which the observer was ‘blind’ to the direction of pattern rotation, to whether it was a treated or untreated *rd1* mouse or age-matched wild-type control animal (C57BL6). The mice were housed in a standard lighting condition for at least 6 h before they were placed in the recording chamber. The observer selected the direction of pattern rotation based on the animal’s optomotor response and the monitors returned to 50% grey until the next trial. Acuity was defined as the highest spatial frequency (at 100% contrast) yielding a threshold response. The measurement was performed using the brightest light condition possible (62 cd m^−2^). Visual acuity was measured in both the eyes of the tested animal and averaged or separately analysed for each eye after four trials were conducted on four separate days. The measurement was carried out on *rd1* mice 11 weeks after treatment together with age-matched isogenic controls. Statistical significance was assessed with a Student’s *t*-test. Results are presented as mean±s.e.m.

## Author contributions

K.M.N. and L.S.C. contributed equally to the concept, design, execution and analysis of all experiments and manuscript writing. MR., K.P., S-M k.H., S.A.A., Y.D., J.R., U.F.O.L. and J.W.B.B. contributed to experimental execution. A.J.S. and R.R.A. contributed to the concept and design of the experiments, funding and to manuscript writing.

## Additional information

**How to cite this article:** Nishiguchi, K. M. *et al*. Gene therapy restores vision in *rd1* mice after removal of a confounding mutation in *Gpr179*. *Nat. Commun.* 6:6006 doi: 10.1038/ncomms7006 (2015).

## Supplementary Material

Supplemsentary Information Supplementary Figures 1-4 and Supplementary Tables 1-2
