# Supplementary material for: Gene therapy restores vision in rd1 mice after removal of a confounding mutation in Gpr179
Source: Nat Commun. 2015 Jan 23;6:6006. doi: 10.1038/ncomms7006 (PMC4354202; doi:10.1038/ncomms7006)
Supplement: Supplemsentary Information — Supplementary Figures 1-4 and Supplementary Tables 1-2 [file ncomms7006-s1.pdf]

Supplementary Figure 1

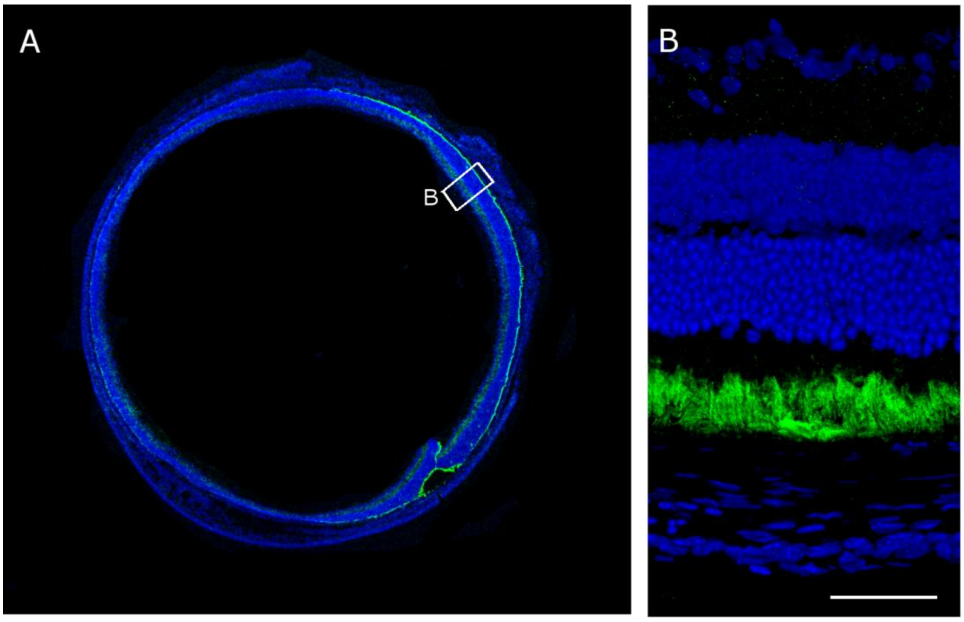

Histological rescue after administration of AAV2/8.hRHO.hPDE6B

Subretinal administration of 1 µL of AAV2/8.hRHO.hPDE6B (5 x 10^{12} vg mL^{-1}) in the *Pde6b*$^{rd1/rd1}$-C3H mouse at P9 results in widespread expression of βPDE protein (green) in the injected hemisphere (A). At two months of age there is a substantial rescue of the outer nuclear layer and inner and outer segment areas (B). Scale bar is 50 µm.

Supplementary Figure 2

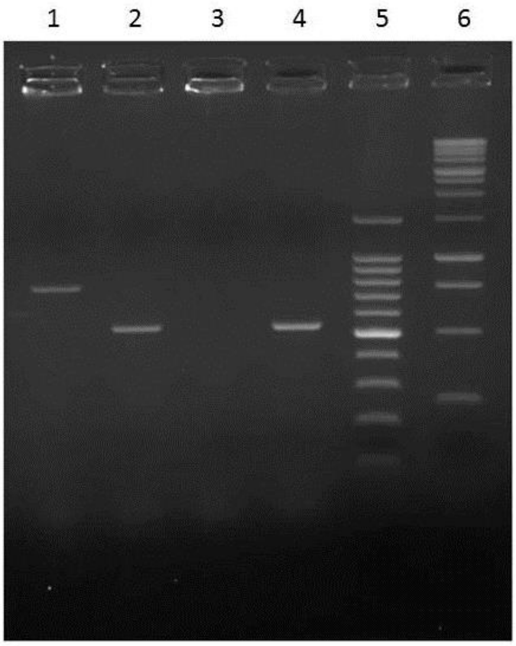

Identification of *Gpr179* mutation in *rd1*-C3H mice from Charles River

PCR analysis of genomic DNA from an *rd1*-C3H mouse purchased from Charles River, using primers for the *Gpr179* wildtype allele (782 bp) and the mutant allele (537 bp). Lane 1: positive control WT allele, Lane 2: positive control mutant allele, Lane 3: *rd1* mouse WT allele, Lane 4: *rd1* mouse mutant allele, Lane 5: 100 bp ladder, Lane 6: 1 kb ladder.

Supplementary Figure 3

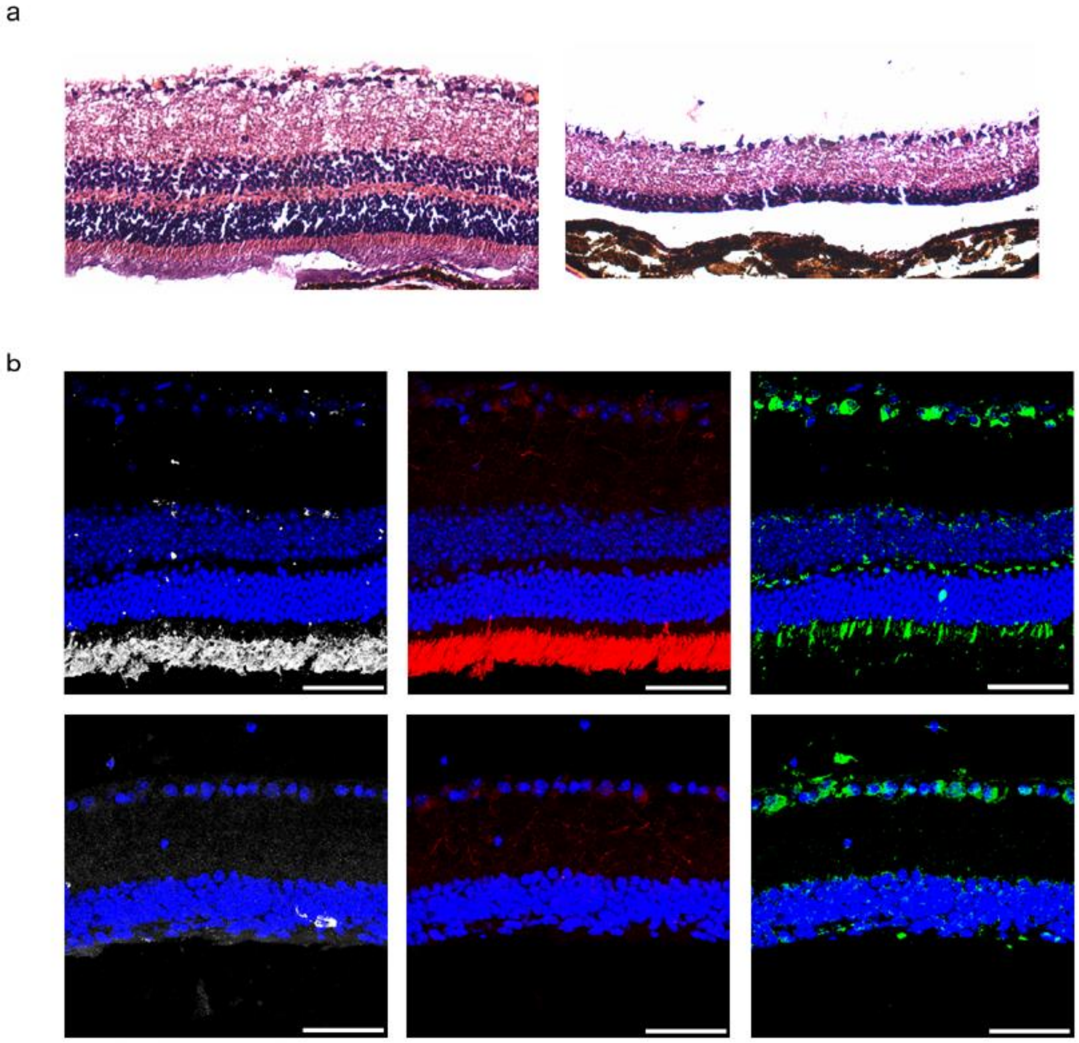

Long term preservation of retinal structure in *Pde6b*$^{rd1/rd1}$-F3 mice

a. Hematoxylin and eosin staining of the retina shows preserved photoreceptor layer in the treated eye (left) whereas the layer is absent in the untreated eye (right) at 13 months after the treatment.

b. Immunohistochemical analysis of the retina shows presence of βPDE encoded by the virally delivered transgene in the outer segments (white; left), rhodopsin also in the outer segments (red; middle), and PNA staining of the cone photoreceptor inner segments and pedicles (green; right) all exclusively in the treated eye (top panels) but not in the untreated eye (bottom panels) at 13 months after the gene therapy. Blue: DAPI staining. Scale bar indicates 50 µm.

Supplementary Figure 4

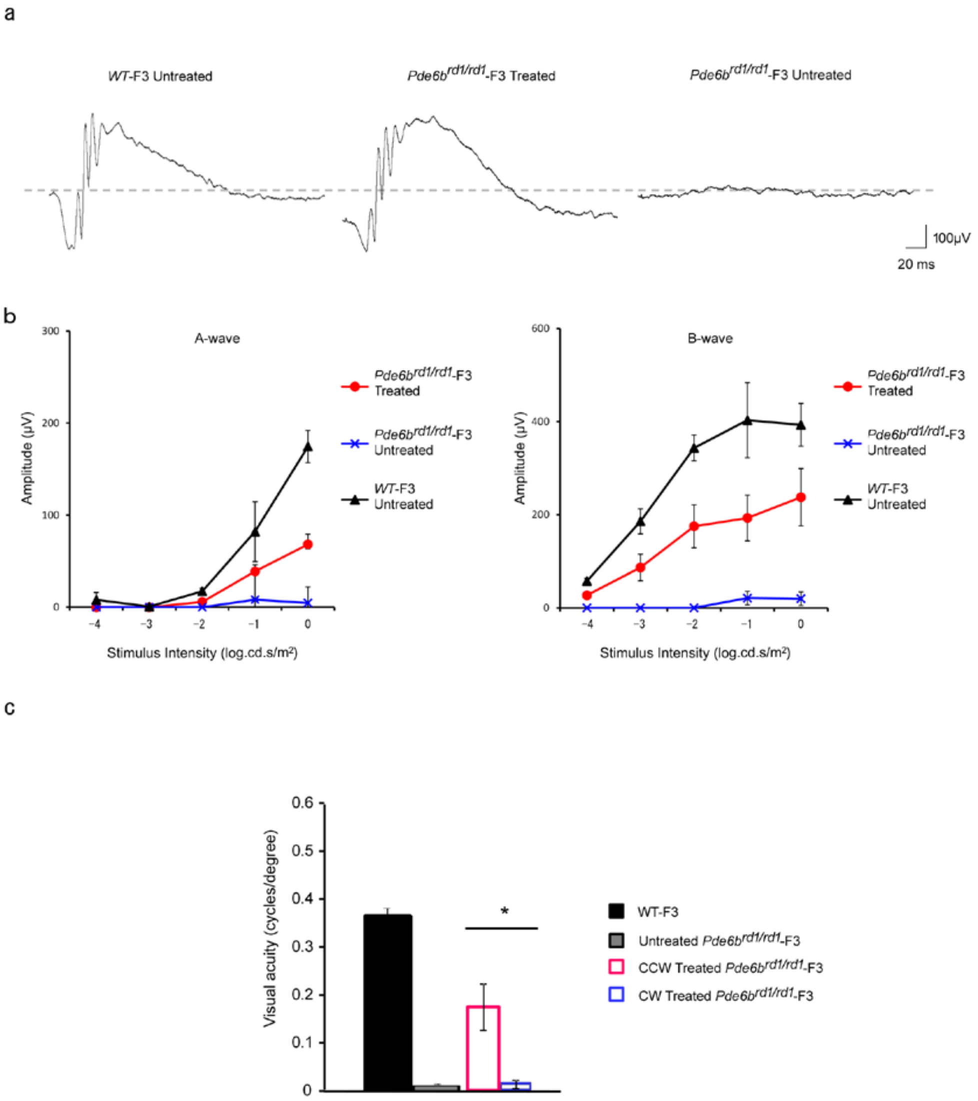

Long term preservation of retinal function in *Pde6b*$^{rd1/rd1}$-F3 mice

a. Representative ERG traces recorded at 11 months after the gene therapy. Right ERG trace is from the untreated eye of the same mouse. Left trace is from age-matched WT-F3 mouse. Scotopic ERGs in response to a 0 log.cd.s/m2 flash are presented.

b. Summary of ERG measurements at 11 months after the gene therapy. Note that the degree of preservation of scotopic retinal responses in the treated eyes (n = 5) compared to the untreated wildtype eyes (n = 3) is similar to the effect size at earlier time points (see Fig. 4c).

c. Preservation of visual acuity 12 months after the gene therapy. The treated eyes had a better acuity compared to the untreated eyes of the same *Pde6b*$^{rd1/rd1}$-F3 mice (n = 5), which were inferior to the age-matched wildtype controls (n = 4). Data represent mean ± S.E.M.

Supplementary Table 1: Sequence of oligonucleotides used for *rd1* and *Gpr179* genotyping.

**Primer**	**Nucleotide sequence (5' – 3')**	**Protocol**	**Fragment size (bp)**
GPR179 F1	CTGGCTGGCTAGTCACAGTCTC	*Gpr179* wt and mutant allele	(see below)
GPR179 R3	CACAGCTGTCAGCGCCATCTTGTAAC	*Gpr179* mutant	537
GPR179 R4	CTTAGCAATGACGCTGGGGATTCAG	*Gpr179* wt	782
RD1 F1	CACGAACACCTGATGGCC	*rd1* point mutation	460
RD1 R1	CATCTTGTCATAGGTGTCTG		

Supplementary Table 2: Cycling conditions for genotyping PCRs

***rd1* genotyping**			***Gpr179* genotyping**		
Time	Temperature	Cycle n^o.	Time	Temperature	Cycle n^o.
3 min	94°C	1X	5 min	95°C	1X
30 sec	94°C	30X	10 sec	95°C	30x
30 sec	54°C		30 sec	61.2^{o}C	
45 sec	72°C		1 min	72°C	
5 min	72°C	1x	5 min	72°C	1x
